# Screening, Purification and Characterization of Protease Inhibitor from *Capsicum frutescens*

**DOI:** 10.6026/97320630014285

**Published:** 2018-06-30

**Authors:** Manju Mohan, Shireen Kozhithodi, Anuraj Nayarisseri, Kothanam Kuzhiyil Elyas

**Affiliations:** 1 Department of Biotechnology, University of Calicut - 673635, Kerala, India; 2 In silico Research Laboratory, Eminent Biosciences, Vijaynagar, Indore - 452010, Madhya Pradesh, India

**Keywords:** Protease Inhibitor, *Capsicum frutescens*, purification, activity

## Abstract

Plants are rich in protease inhibitors (PI) and trypsin inhibitors are the most common. Therefore, it is of interest to screen PI from plant sources. We report the screening, purification and characterization of PI from Capsicum frutescenes. The partially purified PI showed bands corresponding to 21 KDa and was further confirmed using reverse zymography. The enzyme was stable at temperatures below 60°C and a wide range of pH with 65 folds purification. The effect of magnesium ions oxidizing and reducing agents on PI is reported. The large-scale isolation and purification of PI from Capsicum frutescenes is of commercial interest.

## Background

Protease inhibitors (PIs) are peptides or proteins proficient of impeding the catalytic activity of proteolytic enzymes. They can be found in all kingdoms of cellular life including viral genomes, thus exhibiting its wide distribution in nature [1, 2]. The importance of proteolysis in biological processes is reflected by the percentage of genes encoding for proteases and protease inhibitors, accounting for about 2-4% of the genome [3]. These proteases and its inhibitors are known since 20th century and their identification has become more effective because of protease degradomics [4], and association with proteomic tools and enzymatic assays can result in efficient characterization of a number of novel protease inhibitors. Its characterization and exploring its physiological significance has elevated significantly due to their biological applicability in an array of living processes including blood coagulation system, complement cascade, apoptosis, cell cycle and hormone processing pathways [5, 6, 7, 8]. On the other hand, deficiencies or alterations in the regulation of these enzymes determine distinct pathological conditions like cancer, arthritis, neurodegenerative and cardiovascular diseases [9, 10].

PIs (protease inhibitors) are classified based on the protease they inhibit, which comprises of serine, cysteine, aspartic and metalloproteases [11]. Amidst them, serine protease inhibitors are the largest and broadly apportioned superfamily of PIs [1, 12, 13], and on the basis of conserved functional motifs they can be partitioned into many classes, being the Kunitz-type inhibitors the best characterized of them, probably due to their copiousness in several organisms [2, 14, 15, 16, 17]. They play a major part in all organisms by constraining unwanted proteases that may be harmful for its own cells [14]. In humans, they can even control molecular pathways associated with tissue homeostasis, development and cell defense. Some of them are also involved as non-inhibitory molecular chaperons (HSP47), storage (ovalbumin, in egg white), blood pressure regulation (angio tensinogen SERPINA8) and hormone carriage proteins (thyroxine-binding globulin, cortisol-binding globulin) [15, 16]. Therefore, it is of interest to isolate and purify protease inhibitor from Capsicum frutescenes.

Capsicum frutescens, coming under the solanaceae family is a shrubby perennial plant. The leaves are broadly ovate, acuminate, usually wrinkled, and more or less pubescent. It's distributed throughout the warmer parts of India and cultivated. The fruits bear a chemical called capsaicin, which reduces pain sensations. It's associated with an array of medicinal uses including a general ailment for various problems including upset stomach, intestinal gas, stomach pain, diarrhea, and cramps. It can also be used for heart and blood vessels including poor circulation, excessive blood clotting, high cholesterol and prevention of heart diseases. It can also be explored as a relief for toothache, seasickness, alcoholism, malaria and fever.

## Methodology

Leaves of Capsicum frutescens were manually harvested locally and authenticated by Dr. Pradeep, Department of Botany, University of Calicut, Kerala.

### Isolation of PI from Capsicum frutescens

Capsicum frutescens leaves (50g) were collected, washed and homogenized with 0.01 M phosphate buffer, pH 7.5 (200ml) in a blender for 5 minutes. The homogenate was centrifuged at 10000 rpm for 15 minutes at 40C. The crude extract obtained as clear supernatant after centrifugation was used to assay protease inhibitor activity, protein content and specific activity as described.

### Caseinolytic plate assay for PI screening

50 plants were screened for the presence of PI. Casein agar plates were used for initial screening of protease inhibition activity. It is a visualising method for detecting Protease inhibitors.1% Agar and 1% casein was solidified in petri plates. Wells were made on the agar plate using metallic plunger. Central well contains 10μl trypsin (1000 U), surrounding wells contain the plant sample. Plates were kept in incubator at 37°C for overnight. After overnight incubation, adding TCA stopped the reaction and inhibition can be clearly visualized by the decrease in diameter of zone of trypsin in the presence of inhibitor when compared to that of the control plate, which comprised of trypsin alone surrounded by buffer.

### Inhibitory activity assay

To quantify the inhibitory activity, the enzyme assay was performed in presence and absence of extracts using casein as a substrate in 0.1M phosphate buffer as described by Kunitz et al with slight modifications [19] 200μL of trypsin (SRL, India), (0.1 mg/mL) prepared in phosphate buffer was pre incubated with 200 μL of the protease inhibitor at 37°C for 15 minutes. To this mixture 400μL of 1% Hammerstein casein (SRL), prepared in 0.1 M phosphate buffer was added and incubated at 37°C for 30 minutes. The reaction was terminated by the addition of 250μL of 0.44M, trichloroacetic acid (TCA) solution. The reaction mixture was centrifuged at 10,000 rpm for 15 minutes and the absorbance of the clear supernatant was measured at 280 nm in a UV-visible spectrophotometer against appropriate blanks. The TCA soluble peptide fractions of casein formed by the action of trypsin in the presence and absence of the inhibitor were quantified by comparing with tyrosine as standard. One unit of trypsin activity was defined as the amount of enzyme that liberated 1μg of tyrosine per milliliter of the reaction mixture per minute under the assay conditions. One unit of protease inhibitor activity was defined as the decrease by one unit of absorbance of TCA soluble casein hydrolysis product liberated by trypsin action at 280 nm per minute under the assay conditions. For easy computation and understanding the protease inhibitor activity was expressed in terms of percent inhibition of trypsin activity throughout the course of study. Appropriate blanks for the enzyme, inhibitor and the substrate were also included in the assay along with the test.

### Standard purification methods

Ammonium sulphate precipitation of the prepared sample was done (30-90%) [17]. The fractionation using ammonium sulphate precipitation removes unwanted proteins with the simultaneous concentration of the protein of interest. The precipitate obtained was further dialyzed against 0.01M phosphate buffer (pH 7.5) for the removal of ammonium sulphate from the precipitate.

### Purification of PI from Capsicum frutescens

The protease inhibitor fraction obtained after the dialysis of ammonium sulphate precipitation was further purified by ion exchange chromatography using CM cellulose (Sigma-Aldrich) as the cation exchanger. Proteins bind to ion-exchangers due to surface charge. These reversibly adsorbed proteins were eluted by a step gradient of 0.1M NaCl in 0.01 M phosphate buffer with a flow rate of 1ml/min. Peak fractions from the column were pooled and dialyzed against 0.01M phosphate buffer (pH 7.5). The dialyzed fractions were concentrated using amicon UF- 10KDa membrane and assayed for protease inhibitor activity, protein content and specific activity.

### Protein quantification

Protein content was estimated using bovine serum albumin (BSA) as the standard and the concentration was expressed in mg/ml. specific activity of the sample was calculated and expressed as U/mg [18].

### SDS PAGE, Reverse Zymography

The molecular mass and homogeneity was determined by SDSPAGE [20]. Reverse zymography is a gel-based procedure to detect the presence of protease inhibitor. In this method 1% gelatin is used as a substrate for proteolysis and is copolymerized within the polyacrylamide matrix. Protein extracts are fractioned by SDS PAGE and then the gel is treated with 0.1mg/ml trypsin, which degrades gelatin, except in the areas where inhibitory activity is present. After the electrophoretic run, gel was incubated with 2.5% w/v Triton X 100 for 30 min to remove SDS followed by rinsing the gel in phosphate buffer (pH 7.5). After the gelatin hydrolysis, the gel was washed with distilled water and stained. Inhibition of protease activity appears as colored bands against a clear background after staining with coomassie brilliant blue.

### Temperature and pH stability studies

Thermal stability of the inhibitor was studied by incubating purified protease inhibitor (0.1mg/ml) at different temperatures ranging from 40C to 1000C for 60 min. The samples were drawn and protease inhibitor activity of each sample was assessed. The stability of protease inhibitor over a range of pH was determined by performing the inhibitor assay at pH 7.5, after incubating the purified protease inhibitor at different buffers from pH 2-12 for 4hrs at 40C. The different buffers used were HCl-KCl (pH 2), citric acid-sodium citrate (pH 4), phosphate buffer (pH 7.5), Tris (hydroxymethyl) aminomethane-HCl (pH 8), sodium carbonatesodium bicarbonate (pH 10) and potassium chloride-sodium hydroxide (pH 12).

### Effect of metal ions, detergents, reducing and oxidizing agents

Effect of various metal ions on activity of protease inhibitor activity was evaluated by incubating the protease inhibitor along with 1mM concentrations of various metal ions in the inhibitor solution for 30 min followed by measuring the protease inhibitor activity. The metal salts used in the study included sodium chloride, barium chloride, cadmium sulphate, magnesium sulphate, sodium molybdate dehydrate, ferric chloride, calcium chloride dehydrate, mercuric chloride and manganese chloride. Effect of various non- ionic and ionic detergents such as Triton X- 100, Tween-80, Tween-20, SDS and CTAB on protease inhibitor activity was determined by incubating the protease inhibitor in each detergent at 1% w/v for 30 min, dialyzed against 0.01M phosphate buffer pH 7.5 and estimated the residual inhibitory activity. The effect of reducing agents on the activity of protease inhibitor was studied by incubating the protease inhibitor with varying concentrations of β-mercaptoethanol and dithiothreitol for 30 min and measuring the residual inhibitory activities were measured in each concentration of reducing agent. Impact of oxidizing agents on the activity of protease inhibitor was studied by incubating the protease inhibitor with hydrogen peroxide (1- 2%, v/v) and dimethyl sulfoxide (1-5%, v/v) for 30 min and measuring the residual inhibitory activity as described.

### Caseinolytic plate assay

**Figure 1:**
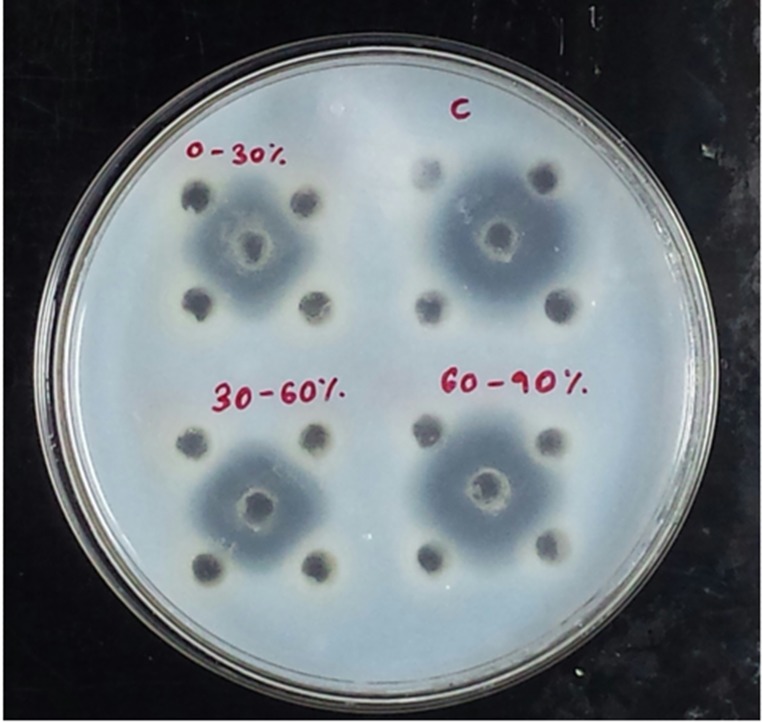
PI on casein agar plates showing zone of inhibition

Out of 18 plants screened for the presence of PI, 11 of them showed trypsin inhibitory activity in the primary screening over casein agar plates (Table 1). Figure 1 shows the inhibition of casein hydrolysis by trypsin by Capsicum frutescens (CF) crude extracts in casein agar plates. Out of 11, CF was showing maximum inhibition and thus was subjected to secondary screening. Tyrpsin alone was showing a visible zone of diameter corresponding to 24 mm.

### Isolation of protease inhibitor

Capsicum frutescens, which showed maximum protease inhibitor activity, was subjected to further screening. Crude samples prepared from the leaves of CF were partially purified by ammonium sulphate precipitation and evaluated for their inhibitory activity. Among the different ammonium sulphate fractions 30-60% fraction was showing maximum specific protease inhibitor activity of 140.67 U/mg (Table 2). Since the 30- 60% fraction recorded maximum protease inhibitor activity, it was selected for the isolation of protease inhibitor and further studies.

### Purification of the protease inhibitor

**Figure 2:**
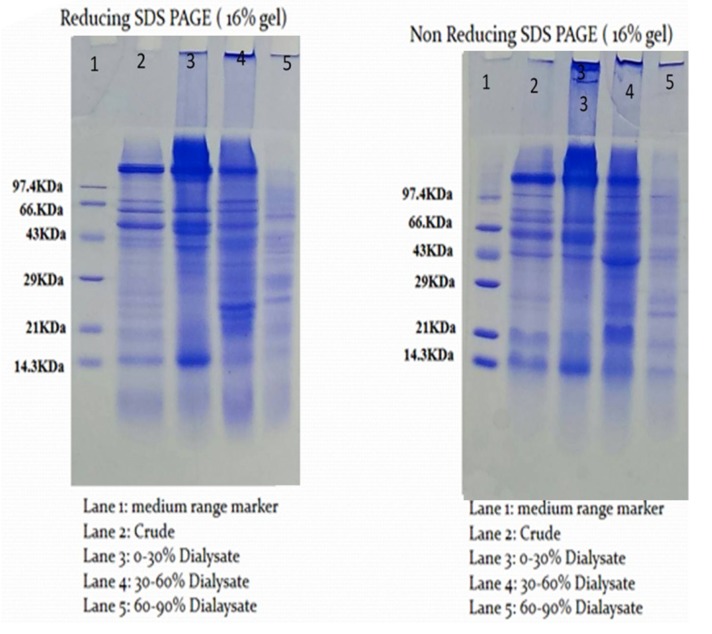
SDS PAGE profile of the ammonium sulphate fractions

Standard purification methods were employed for the purification of protease inhibitor. The crude extract was purified employing ammonium sulphate precipitation, dialysis and CM cellulose chromatography. The yield and fold of purification of protease inhibitor extracted from the leaves of Capsicum frutescens is summarized in Table 3. The dialysate obtained after ammonium sulphate saturation (30-60% fraction) was subjected to ion exchange chromatography using CM cellulose. 2ml (5.6 mg) of ammonium sulphate (30-60%) fraction was loaded to CM cellulose column (15x1.5 cm). peak fractions were pooled and concentrated using Amicon 10 KDa membrane. Inhibitory activity was shows peak with 65.81 fold of purification (Figure 2).

### Characterization of the protease inhibitor

**Figure 3:**
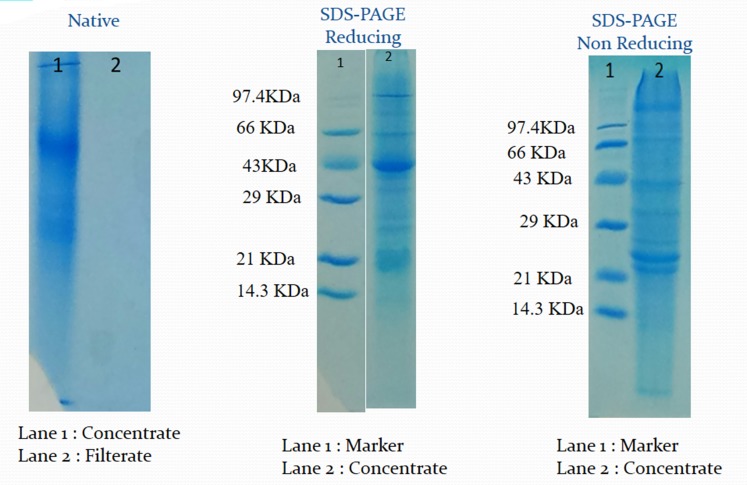
SDS PAGE profile after CM cellulose chromatography purified fraction

**Figure 4:**
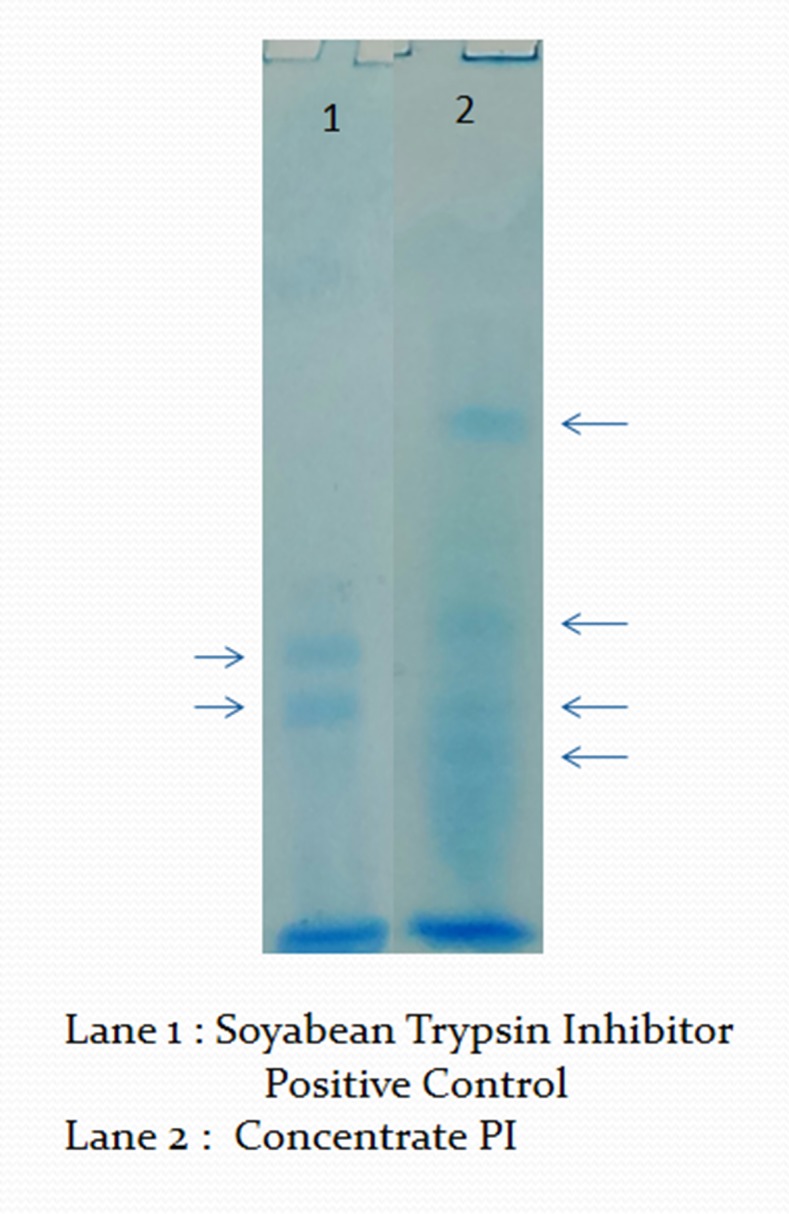
Reverse zymogrphy of the PI

Partially purified inhibitor was further subjected to characterization for its biophysical and physicochemical properties like molecular weight, stability at different temperature and pH. SDS PAGE profile of the peaks is depicted in Figure 3. Both reducing and non-reducing SDS-PAGE was performed. The molecular weight from the non-reducing SDSPAGE was determined to be 43 KDa, the prominent band present and which gets cleaved in the reducing SDS-PAGE into two bands of molecular weight less than 21 KDa each. This indicates that the PI is a protein of 43 KDa and is a dimer with molecular weight less than 21KDa (Figure 3). This was further confirmed by reverse zymography, which is done for confirming the presence of protease inhibitor. Blue bands on lighter background confirmed the presence of protease inhibitor (Figure 4).

### Stability studies

**Figure 5:**
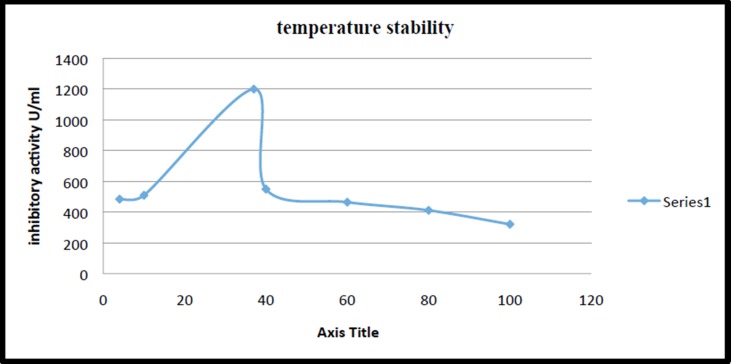
Effect of temperature on PI activity

**Figure 6:**
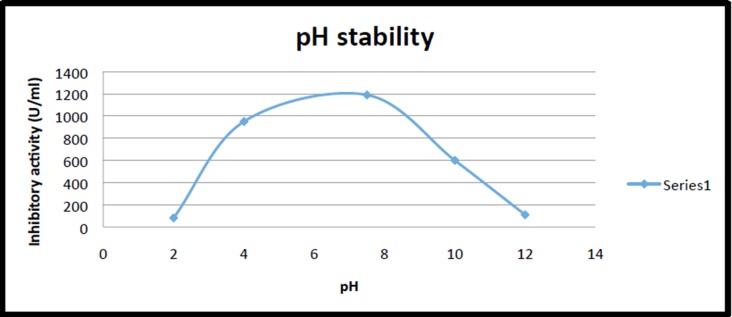
Effect of pH on PI activity

The partially purified Capsicum frutescens protease inhibitor (CFPI) was observed to show considerable stability over a broad range of temperatures up to 90°C. Although maximum activity was observed around 30-40°C which gradually decreased (Figure 5). These observations indicate that the PI has high intrinsic stability and which is a desirable trait for most of the biotechnological applications of proteins and for their commercial exploitation [21]. Similarly considerable stability was observed for a pH range up to 12, the maximum inhibitor activity was observed at pH 7.5. It was noted that at high acidic condition of pH 2-4, the PI was unstable (Figure 6). The high thermal and pH stabilities of inhibitor have wide application in various industries.

### Effect of metal ions, reducing and oxidizing agents

**Figure 7:**
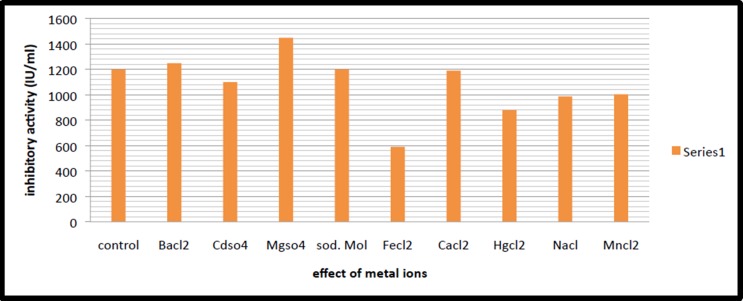
Effect of metal ions on PI activity

**Figure 8:**
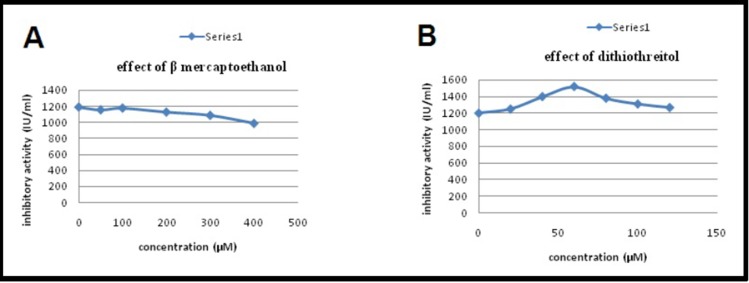
Effect of reducing agent (β mercaptoethanol) (A) and reducing agent (dithiothreitol) (B) on PI activity

Ca2+, Mg2+, Mn2+, Hg2+, Na2+, Ba2+, Cd2+, Fe3+, Mo6+ were studied for their impact on the activity of CFPI. Addition of Mg2+ at 1mM concentration led to an enhancement in the PI activity (Figure 7). None of the divalent ions could enhance PI activity. The effect of different concentrations of β-mercaptoethanol and dithiothreitol was studied and the results are documented in Figure 8.

**Figure 9:**
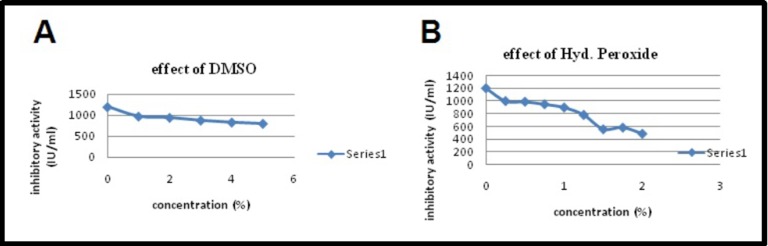
(a) Effect of oxidizing agent (DMSO) on inhibitory activity of PI; (b) Effect of oxidizing agent (H2O2) on PI activity

From the results it was inferred that the activity was increased by dithiothreitol up to a concentration of 60μM, and a concentration above 140μM resulted in the complete inactivation of the inhibitor. However, in the case of β - mercaptoethanol inactivation occurred at a concentration of 400μM. The results depicted in Figure 9 testify that the protease inhibitor activity decreased along with increase in concentrations of oxidizing agents H2O2 and DMSO.

## Discussion

Proteolysis is one of the most indispensable metabolic processes entailed for protein processing and turnover. During germination, proteases are involved in the degradation of storage proteins for the assimilation of nitrogen into biosynthetic pathways. These enzymes are indispensable in developmental processes like programmed cell death. Proteases are highly specific to their substrate, and the specificity depends on the localization of the substrate and the proteolytic enzyme, and structural and chemical properties at the active site of the enzyme [22]. Plants are a rich source of protease inhibitors. In this context in the present study, plants were screened based on the activity of protease inhibitor and thus as a potential source for deriving protease inhibitor. Among the different plants screened subjected to partial purification with ammonium sulphate saturation, maximum activity was demonstrated by Capsicum frutescens extract compared to the others which showed much lesser activity. Hence C. frutescens was selected as the potential source.

The proteinaceous nature of the PI was confirmed further after partial purification using ammonium sulphate precipitation. The phosphate buffer of pH 7.5 was standardized for the extraction of PI and it was reported to be a good extractant for the maximal extraction of proteins from Cajanus cajan seeds and Moringa oleifera with high amount of trypsin inhibitor activity and protein concentration [23, 24]. The optimum pH for protease activity was 7.5.

Capsicum, also known as red pepper or chilli pepper, is an herb. The fruit of capsicum plant is used to make medicine and is used as a spice in food worldwide. The principal constituent being capsaicin, seems to reduce pain sensations when applied to the skin. This compound is responsible for its pharmacological properties especially against obesity, hyperglycemia and pain [25].

The inhibitory protein obtained from C.frutescens was partially purified by ammonium sulphate precipitation, followed by CM cellulose chromatography. It was found that 30-60% ammonium sulphate saturation was efficient for precipitating the protease inhibitor compared to other fractions. The fold of purification of protease inhibitor obtained for ion exchange chromatography was 65.81 times. Fold and recovery of protein can be increased by the combination of purification methods. The same results were also reported in other plant species e.g.chickpea [26], A.senegal [27], and A.nilotica [28]. The fold of purification obtained in this study is comparatively higher when compared to other species [29, 27, 28]. Protease inhibitor purification methods reported in the research on legumes, achieved purification levels, which ranged from 19 to 489. The purification folds were 489 for kidney bean [30], 19 for P.mungo [31], 116.2 for Dimorphandra mollis, 246 for Prosopis juliflora [33], and 29 for Terminalia arjuna [34].

The purification steps were observed in 12.5% SDS-PAGE and resolved in protein bands. The size of the plant protease inhibitor varied from 4 to 85 KDa with the majority in the range of 8-20 KDa [35]. Different molecular mass was reported for protease inhibitor from different plant resources e.g. cowpea (18.5 KDa), soybean (19 KDa), mustard seed (20 KDa) and Cajanus cajan (14 KDa) [36, 37, 38].

Most of the plant inhibitors are active at temperatures upto 50°C. In the present study the inhibitor was showing wide temperature stability. The stability at high temperature of the inhibitor may be attributed to its rigid and compact protein structure stabilized by a number of disulphide linkages, as suggested for protease inhibitor from pea seeds [39]. Similarly all the protease inhibitors isolated from plants have a wide range of pH from 2 to 10 [23]. The maximum activity was at pH 7.5, while the inhibitors were unstable at extreme acidic pH (pH 2). The amino acid composition of plant protease inhibitors is enriched in cysteine residues that are significant in the formation of disulphide bridges and in conferring stability to heat, pH changes, and proteolysis [35].

In the present study among the metal ions evaluated for their impact on the activity of protease inhibitor. Addition of Mg2+ at 1mM concentration, led to an enhancement in the protease inhibitor activity. None of the other divalent ions could enhance the activity. Involvement of heavy metal ions in the activity of cysteine proteases, papain and clostripain has been reported earlier [40]. These ions may be involved in maintaining structural integrity of the inhibitor. Results obtained from the effect of oxidizing agents H2O2 and DMSO on PI showed that the inhibitory activity decreased along with an increase in concentrations of oxidizing agents. Further increase in concentration led to complete inactivation of the same. Similarly, inhibitory activity of PI isolated from M.oleifera was declined in response to an increase in the concentration (from 1% to 5%) of oxidizing agents H2O2 and DMSO [23]. Intra sulfide bonds are vital for the proper folding and stability of many proteins. The effect of reducing agents studied on PI activity using β- mercaptoethanol and dithiothreitol indicated complete inactivation of the inhibitor at concentrations above 150 μM of dithiothreitol. Compared to this, β-mercaptoethanol inactivation was noted at 400μM.

## Conclusion

A protease inhibitor from Capsicum frutescenes was isolated and partially purified. The physical and chemical characterization shows that PI was stable over a wide range of temperature and pH. Large-scale isolation of PI is of commercial interest.

## Figures and Tables

**Table 1:** Primary screening of plants for the presence of PI on casein agar plates

Sl. no	Name of the plant	Diameter of zone
1	*Pinenta dioica*	19
2	*Bongainvillea spectabilis*	18
3	*Excoecaria cohinschinesis*	20
4	*Gliricidia sepium*	22
5	*Hydrangea macrophylla*	18
6	*Sypgium cumini*	20
7	*Manilkara zapota*	17
8	*Coccinia grainds*	20
9	*Curcuma longa*	20
10	*Acaciaa mangium*	19
11	*Piper nigrum*	18
12	*Carika papaya*	19
13	*Coleus amboinicus*	19
14	*Myristica fragrans*	18
15	*Ocimum sanctum*	20
16	*Costus pictus*	19
17	*Ixora polyantha*	18
18	*Capsicum frutescenes*	15

**Table 2:** Specific activity of ammonium sulphate fractions

Ammonium sulphate (%)	Total volume (ml)	Protein concentration (mg)	Inhibitor activity on agar plates (mm)	Total activity (U)	Specific activity (U/mg)
Crude	200	880	16	90240	102.58
0-30%	10	51	16	3311	64.92
30-60%	10	52	14	7315	140.67
60-90%	10	32	17	889	27.78

**Table 3:** Yield of protein, Yield of protease inhibitor activity and Fold of purification in comparison with crude extract.

Purification method	Volume (ml)	Total protein (mg)	Total activity (U)	Specific activity (U/mg)	Yield of protein	Yield of activity	Fold purification
Extract	200	880	90240	102.54	100*	100*	1*
(NH4)2SO4	10	52	7315	140.67	6	8.106	1.37
CM cellulose	10	12	8098	6749	0.136	7.47	65.81
